# Evaluation of fluid status in patients with acromegaly through bioelectrical impedance vector analysis: a cross-sectional study

**DOI:** 10.1007/s40618-025-02541-4

**Published:** 2025-02-15

**Authors:** Emanuele Varaldo, Nunzia Prencipe, Alessandro Maria Berton, Daniela Cuboni, Luigi Simone Aversa, Michela Sibilla, Francesca Mocellini, Fabio Bioletto, Ezio Ghigo, Valentina Gasco, Silvia Grottoli

**Affiliations:** Division of Endocrinology, Diabetology and Metabolism, Department of Medical Sciences, University of Turin, Turin, Italy

**Keywords:** BIVA, IGF-I, GH, Total body water, Extracellular fluid, Overhydration

## Abstract

**Purpose:**

The acromegalic state is associated with an increase in total body water and sodium. The aim of our study was to assess the hydration status of patients with acromegaly using bioimpedance vector analysis (BIVA), differentiating patients according to their disease status (active, medically controlled or cured) and to compare the confidence and tolerance ellipses of BIVA in those patients in relation to a reference healthy population.

**Methods:**

We analyzed data from 73 consecutive patients aged 18 years or older, diagnosed with acromegaly and undergoing regular follow-up at our Division for whom a BIVA analysis was available. Patients were evaluated through BIVA and insulin-like growth factor I (IGF-I), growth hormone (GH), serum sodium and potassium, creatinine, glucose, HbA1c and plasma and urine osmolality were collected. Exclusion criteria were concurrent presence of arginine-vasopressin deficiency, dysnatremia or the presence of pathologies known to significantly alter the extracellular fluid.

**Results:**

Sixty-nine patients (M/F 34/35, age 60 ± 14 years) were enrolled in the study. As expected, patients with active disease (*n* = 22) presented higher IGF-I and GH levels compared to other subjects. Patients with controlled disease (*n* = 33) were significantly older than other individuals (*p* = 0.028 vs. active disease, *p* = 0.024 vs. cured disease). Compared to a reference healthy population, patients with either active or medically controlled disease showed significant fluid overload (*p* < 0.0001 for both males and females) and BIVA confidence analysis demonstrated that there were no significant differences in hydration status between the two groups (*p* = 0.363). On the other hand, patients with cured disease (*n* = 14) showed reduced hydration status compared to patients with active disease (*p* = 0.016), although no difference was observed compared to patients with controlled disease (*p* = 0.308).

**Conclusion:**

The results of our study demonstrate that patients with either active or medically controlled acromegaly present a significant overhydration compared to a healthy reference population and that alterations in body water content usually improve in individuals with cured disease.

**Supplementary Information:**

The online version contains supplementary material available at 10.1007/s40618-025-02541-4.

## Introduction

Acromegaly is an insidious disease resulting from an overproduction of growth hormone (GH) and elevated levels of insulin-like growth factor I (IGF-I), generally caused by a functioning pituitary adenoma [1].

The hypersecretion of GH/IGF-I in subjects with acromegaly is responsible for numerous adverse outcomes, with metabolic and cardiovascular complications being typically responsible for the increased mortality observed in these patients [2, 3]. Of note, various medical treatments have reported benefits regarding these disease complications [4, 5, 6, 7], but it is not clear if acromegaly patients may still experience increased long-term mortality despite good disease control compared to the general population [8].

Among the various systemic effects mediated by the somatotropic axis, in particular, the sodium and water-retaining effect has been recognized for decades [9], although the pathophysiological mechanism has not been fully elucidated yet. Acromegaly patients usually present an increase in total body water (TBW) and extracellular fluid (ECF) which contributes to several clinical complications of the disease, including acromegalic hypertension and cardiomyopathy, carpal or cubital tunnel syndrome and obstructive sleep apnea syndrome [10].

The primary treatment for most acromegaly patients is surgical, as surgery constitutes the only possibility of a definitive cure in the short-term, leading to immediate lowering of GH levels and rapid enhancements in cardiovascular, respiratory, and endocrinological functions [11]. In this regard, after successful resection of a GH-secreting adenoma, an immediate amelioration in soft-tissue edema has been reported in over 90% of patients, as a result of the increased diuresis secondary to rapid GH depletion [12]. Indeed, it has been recently suggested that a negative fluid balance in the first 48 h after surgery in the absence of arginine vasopressin (AVP) deficiency may be associated with long term remission [13].

To date, however, most studies regarding the hydration status of acromegaly patients are outdated. The gold standard for assessing body water remains isotope dilution [14, 15], but this method exposes patients to radiation and entails high costs, and therefore its use is limited to research settings only. In this context, bioelectrical impedance vector analysis (BIVA) is a tool that allows a reliable and reproducible assessment of the distribution of body fluids in several clinical settings [16, 17, 18], with the ability to compare the hydration status of the subject with that of a healthy control population of the same sex [19, 20]. The BIVA technique utilizes bioelectrical impedance analysis (BIA), which refers to the safe and non-invasive measurement of an organism’s passive electrical properties following the introduction of a painless, low-level alternating current into the body [16].

As of today, only few studies are available regarding the evaluation of bioimpedance characteristics in acromegalic patients, all involving small cohorts [21, 22, 23]. Taking this into account, the aim of our study was to assess the hydration status of patients with acromegaly through BIVA, according to their disease status (either active, medically controlled or cured).

## Materials and methods

All patients aged 18 years or older, diagnosed with acromegaly and undergoing regular follow-up at the Division of Endocrinology, Diabetology and Metabolism of the University Hospital “Città della Salute e della Scienza di Torino” (Turin, Italy) were screened.

Patients were included in the study if a BIVA analysis performed on the same day as the disease monitoring exams was available. On the other hand, exclusion criteria were: (1) concurrent presence of AVP deficiency, as a significant confounder of hydration status; (2) detection of hypotonic hyponatremia or hypernatremia at the biochemical evaluation performed on the same morning of the BIVA analysis; (3) the presence of concurrent pathologies known to significantly alter the ECF (i.e., heart failure with ejection fraction < 40%, chronic kidney disease with estimated glomerular filtration rate (eGFR) < 30 ml/min/1.73m^2^, or severe liver failure with ascites).

Demographic and clinical details, including the characteristics of pituitary adenomas and disease-related information were collected. Moreover, information regarding reported salt and water intake was recorded through direct inquiry.

All BIVA analyses were conducted between 07:00 and 09:00 in a quiet room at fasting state at the endocrine investigation day unit. The morning of the test, data on resistance (Rz), reactance (Xc), phase angle (PhA), weight (kg), height (cm), body mass index (BMI, kg/m^2^), blood pressure (mmHg) and heart rate (beats per minute) were collected. Moreover, a blood sample for IGF-I, GH, serum sodium (s-Na), serum potassium (s-K), blood glucose (BG), HbA1c, plasma osmolality (p-Osm), creatinine and a urine sample for urine osmolality (u-Osm) were collected.

Acromegaly was diagnosed according to current guidelines and patients were later classified according to hormonal status as active (IGF-I > 1xULN [upper limit of normality]) or controlled (IGF-I ≤ 1xULN) disease [11]. Similarly, patients with cured acromegaly were defined by the presence of IGF-I levels within the normal range for age at evaluation performed 12 months after either neurosurgery or discontinuation of therapy, in the absence of any ongoing medical treatment [11].

Any concomitant hormonal deficits were treated with specific replacement therapy at an adequate dose, with the exception of growth hormone deficiency (GHD). In fact, in patients with cured acromegaly and normal IGF-I values, the possible presence of GHD in the postoperative period was not assessed by dynamic test, following the clinical practice of our center. However, also in patients with cured acromegaly and three other documented pituitary hormone deficits, in which GHD is strongly suspected [24], no rhGH replacement therapy was administrated.

The study was approved by the Local Ethics Committee (cod. 0040828) and was in accordance with the principles of the Declaration of Helsinki. Written informed consent was obtained from all study participants.

### Laboratory measurements

Serum GH levels (µg/L) were measured by automated (LIAISON^®^ analyzer) one step quantitative chemiluminescent immunoassay (CLIA) (Liaison hGH, Diasorin). The CLIA assay of GH is a sandwich-type assay and utilizes two monoclonal antibodies directed against the 22 KDa form of the GH peptide. Calibrators are balanced out on the international standard WHO 2nd IS 98/574 in human serum. The sensitivity of the assay was 0.052 µg/L while the inter- and intra-assay coefficients of variation (CV) were 5.0–6.0% and 4.0–5.0%, respectively. GH levels were not assayed in subjects undergoing therapy with pegvisomant.

Serum IGF-I levels (µg/L) were measured by automated (LIAISON^®^ analyzer) one step quantitative CLIA (Liaison IGF-I, Diasorin) after acid-ethanol extraction, to avoid interference by binding proteins. The CLIA assay of IGF-I is a sandwich-type assay and utilizes two monoclonal antibodies. The sensitivity of the method was 3 µg/L while the inter- and intra-assay CV were 6.0–10.0% and 3.0–5.0%, respectively.

Considering the variability in normal values based on patients’ age, IGF-I levels were additionally standardized according to ULN.

All other biochemical variables were assayed in serum, plasma or urine samples according to the automated methods currently used in the analysis laboratory of our center.

## BIVA

BIVA was evaluated by an impedance vector analyzer with measurement frequency of 50 kHz ± 1% (BIA101BIVA^®^, Akern, Loc. Montacchiello, Pisa, Italy) [17, 18]. The 50 kHz frequency is highly accurate for evaluating the ECF, as it does not penetrate the intracellular compartment [25].

In terms of bioimpedance parameters, Rz reflects conductivity through ionic solutions and inversely correlates with the body’s water and electrolyte content, while Xc represents impedance related to the membrane capacitance of metabolically active cells. PhA, a derived parameter, indicates the ratio between intracellular and extracellular fluid volumes and is considered a marker of cellular health [26]. Both Rz and Xc were normalized according to patient height (H) and plotted on a Rz/H and Xc/H graph (Biavector, Bodygram ­ Plus^®^ version 1.31) [27]. The vector’s position and magnitude within this plot offer valuable insights into hydration status, body cell mass, and cellular membrane integrity. The vector’s orientation, defined by PhA, represents the geometric relationship between Rz and Xc. Lateral vector shifts, caused by variations in reactance, reflect changes in dielectric mass, which correlate with the quantity of cellular membranes and tissue interfaces within soft tissues. The vector’s length serves as an indicator of hydration status: a shortened vector indicates fluid overload due to decreased resistance, while a lengthened vector suggests dehydration as resistance increases [16, 26].

Furthermore, Biavector allows to compare the variations between repeated measurements on the same subject with the normal sex-specific ellipses of the general healthy population [19, 25]. Reliable thresholds for both overhydration and dehydration conditions have been previously identified at the lower and upper poles of the 75th sex-specific tolerance ellipse, respectively [19]. Similarly, BIVA confidence analysis enables the comparison of ellipses across different populations to identify differences in vector position and, consequently, in hydration status [27]. In this regard, the definition of over/dehydration was based on the presence of a significant displacement of the ellipses of the groups being compared and the greater or lesser length of the Biavector. The estimates of TBW, ECF, and intracellular fluid (ICF) were derived using proprietary algorithms provided by the manufacturer (Bodygram Plus^®^ version 1.31).

For BIVA analysis, patients were instructed to remove any metal objects before the procedure and then lie supine on a non-conductive medical surface and they remained in this position for a sufficient rest period to allow for uniform distribution of body fluids. Two distal current-introducing electrodes were placed on the dorsal surfaces of the hand and foot proximal to the metacarpophalangeal and metatarsophalangeal joints, respectively. In addition, two voltage-sensing electrodes were applied at the pisiform prominence of the wrist and between the medial and lateral malleoli of the ankle [28]. Distance between electrodes pair was 5 cm.

### Statistical analysis

Normally and non-normally distributed variables were expressed as mean and standard deviation (SD) or median and interquartile range (IQR), respectively, while categorical data were expressed as counts and percent. Normality was assessed using the Shapiro-Wilk test. Differences between groups were evaluated by Student’s t-test for independent samples in the case of variables with normal distribution; to highlight the differences between the median values of non-normally distributed variables Mann-Whitney test was used when appropriate. The chi-square test and the Fisher’s exact test were used to evaluate the association between binary variables, while the Spearman’s test to evaluate the correlation of continuous ones. With respect to BIVA, mean Biavectors’ displacement were compared with the two samples Hotelling’s T2 test [20].

A cut-off of p-value < 0.05 was considered statistically significant. Statistical analysis was performed using MedCalcTM^®^ (Statistical Software version 20.007, MedCalc Software Ltd, Ostend, Belgium). BIVA analyses were made using BIVA 2002^®^ software (Microsoft, Padova, Italy) [20].

## Results

Between December 2022 and June 2024, 148 patients with acromegaly were evaluated at our Division. BIVA analysis was available in 73 patients, and among them 4 patients were subsequently excluded: 2 patients presented concurrent AVP deficiency, 1 patient was affected by severe renal insufficiency (eGFR < 30 ml/min/1.73m^2^), and 1 patient was excluded due to evidence of hypotonic hyponatremia.

**Fig. 1:**
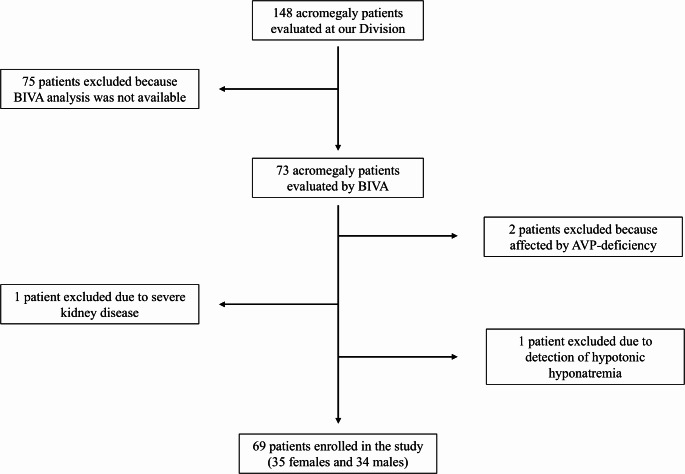
Enrollment process flowchart. BIVA: bioimpedance vector analysis; AVP: arginine-vasopressin

Eventually, 69 patients (34 males and 35 females) were included in the study (Fig. 1).

## Patient characteristics

The anamnestic and clinical characteristics of the patients enrolled in the study are presented in Tables 1 and 2. In all subjects, the reported mean daily water intake was normal (< 3 L/day, range 1–2.5 L/day), as was the dietary salt intake (3–5 g/day). Additionally, no patient reported the use of non-steroidal anti-inflammatory drugs [17] in the week preceding the morning of the examination.

**Table 1:** Demographic and clinical characteristics of the patients enrolled in the study. Data are expressed as mean ± standard deviation (SD) or median and interquartile range (IQR) or n (%). The numbers in bold indicate significant values ( p < 0.05). BMI: body mass index; SRL: somatostatin receptor ligand

Patients’ characteristics	Overall (*n* = 69)	Active disease (*n* = 22) (a)	Controlled disease (*n* = 33) (b)	Cured disease (*n* = 14) (c)	(a) vs. (b) *p*-value	(a) vs. (c) *p*-value	(b) vs. (c) *p*-value
Sex, *female (%)*	35 (50.7)	12 (54.5)	16 (48.5)	7 (50)	0.663	0.793	0.925
Age, *years*	60 ± 14	56 ± 16	64 ± 13	56 ± 9	**0.028**	0.988	**0.024**
Height, *cm*	170 ± 11	169 ± 11	169 ± 11	173 ± 9	0.861	0.244	0.266
Weight, *kg*	76.7 ± 15.0	78.5 ± 15.1	74.2 ± 15.0	79.5 ± 15.4	0.308	0.845	0.279
BMI, *kg/m*^*2*^	25.7 (24.0–28.5)	26.5 (24.8–30.0)	25.5 (23.5–28.5)	25.3 (23.0–27.4)	0.216	0.269	0.889
Systolic blood pressure, *mmHg*	130 (114–140)	130 (115–145)	130 (110–140)	123 (115–140)	0.917	0.719	0.691
Diastolic blood pressure, *mmHg*	80 (70–85)	78 (70–85)	75 (68–80)	80 (70–85)	0.130	0.691	0.102
Heart rate, *bpm*	74 ± 10	76 ± 7	73 ± 10	75 ± 12	0.255	0.933	0.451
Obesity, *n (%)*	12 (17.4)	6 (27.3)	4 (12.1)	2 (14.3)	0.175	0.441	0.841
Diabetes mellitus, *n (%)*	21 (30.4)	8 (36.4)	13 (39.4)	0 (0)	0.822	**0.013**	**0.005**
Arterial hypertension, *n (%)*	29 (42.0)	10 (45.5)	16 (48.5)	3 (21.4)	0.827	0.149	0.087
Disease duration, *years*	12.0 (4.5–19.0)	4.9 (0.6–15.0)	14.0 (9.0–21.0)	**-**	**0.004**	**-**	**-**
Remission duration, *years*	-	-	-	7 (4–9)	**-**	**-**	**-**
Adenoma’s diameter at the last follow-up, *mm* *(among adenomas; n = 42)*	8 (5–12)	8 (5–12)	7 (5–15)	-	0.990	-	-
Previous neurosurgery, *n (%)*	41 (59.4)	13 (59.1)	16 (48.5)	12 (85.7)	0.444	0.142	**0.024**
Previous radiotherapy, *n (%)*	9 (13.0)	2 (9.1)	5 (15.2)	2 (14.3)	0.513	0.633	1.000
No ongoing medical therapy, *n (%)*	24 (34.8)	10 (45.5)	0 (0)	14 (100)	**< 0.001**	**< 0.001**	**< 0.001**
Cabergoline therapy, *n (%)*	11 (15.9)	2 (9.1)	9 (27.3)	-	0.168	-	-
SRL therapy, *n (%)*	41 (59.4)	11 (50)	30 (90.9)	**-**	**< 0.001**	**-**	**-**
Pegvisomant therapy, *n (%)*	10 (14.5)	3 (13.6)	7 (21.2)	-	0.723	-	-
Association therapy, *n (%)*	14 (20.3)	3 (13.6)	11 (33.3)	-	0.125	-	-
Central adrenal insufficiency, *n (%)*	7 (10.1)	2 (9.1)	4 (12.1)	1 (7.1)	1.000	1.000	1.000
Central hypothyroidism, *n (%)*	12 (17.4)	3 (13.6)	6 (18.2)	3 (21.4)	0.727	0.658	1.000
Hypogonadotropic hypogonadism, *n (%)*	15 (21.7)	4 (18.2)	9 (27.3)	2 (14.3)	0.528	1.000	0.463
Anterior panhypopituitarism, *n (%)*	3 (4.3)	0 (0)	1 (3.0)	2 (14.3)	1.000	1.000	1.000

Patients with active disease were younger (56 ± 16 vs. 64 ± 13 years, *p* = 0.028) and with a shorter disease duration (4.9 [0.6–15.0] vs. 14.0 [9.0–21.0] years, *p* = 0.004) compared to patients with medically controlled disease, while no differences regarding age were evidenced compared to patients with cured disease. Ten patients with active disease were assessed upon acromegaly diagnosis, and consequently had no ongoing medical therapy. No difference was observed in the frequency of arterial hypertension and obesity among the three groups, though a tendency towards significance was noted regarding the rate of hypertension between patients with cured and controlled acromegaly, higher in the latter group. Conversely, cured patients were significantly less affected by diabetes mellitus.

**Table 2:** Antihypertensive and antidiabetic therapy in the cohort of acromegalic patients. ACE-i: angiotensin-converting enzyme (ACE) inhibitor; ARB: angiotensin II receptor blockers; CCB: calcium channel blockers, DPP IV: dipeptidyl peptidase IV; GLP-1RA: glucagon-like peptide-1 receptor agonist; SGLT-2i: sodium-glucose transport protein 2 inhibitor

Anti-hypertensive drugs (among hypertensive patients)	Overall (*n* = 29)	Active disease (*n* = 10)	Controlled disease (*n* = 16)	Cured disease (*n* = 3)	*p*-value
ACE-I/ARB, *n *	25	7	15	3	0.178
CCB, *n *	9	3	5	1	0.994
Thiazide diuretics, *n *	5	0	4	1	0.192
Loop diuretics, *n *	3	1	2	0	0.808
Beta-blockers, *n *	9	3	4	2	0.358
**Anti-diabetic treatment** **(among diabetic patients)**	**Overall** ***(n = 21)***	**Active disease** ***(n = 8)***	**Controlled disease** ***(n = 13)***	**Cured disease** ***(n = 0)***	
Nutrition therapy only, *n *	7	3	4	-	1.000
Metformin monotherapy, *n *	5	2	3	-	1.000
Metformin + DPP IV inhibitors / GLP-1RA / long-acting insulin, *n *	4	2	2	-	0.400
SGLT-2i monotherapy, *n *	1	1	0	-	0.381
SGLT-2i association therapy, *n *	4	0	4	-	0.131

Forty-one patients (59.4%) had previously undergone neurosurgery (108 [52–185] months before), and 9 subjects had also received adjuvant radiotherapy (141 [105–304] months before). Among patients undergoing medical therapy with somatostatin receptor ligands (SRLs, *n* = 41), 37 patients were on treatment with first-generation SRLs (21 patients on lanreotide and 16 patients on octreotide) and 4 patients were on treatment with pasireotide. In 13 cases (23.6%), despite evidence of persistent somatotropic hypersecretion, the presence of adenomatous tissue was no longer visible (empty sella was observed in 7 subjects and a normal pituitary morphology was appreciated in 6 patients).

As expected, patients with controlled acromegaly disease had significantly lower levels of IGF-I (*p* < 0.001) and GH (*p* = 0.001) compared to patients with active disease while no difference was observed compared to patients with cured disease. No difference was observed in relation to the other biochemical parameters, with the exception of BG levels and HbA1c, which were lower in patients with cured disease (Table 3). Regarding antidiabetic treatment, no patient was treated with rapid-acting insulin, and only two patients were treated with long-acting insulin in combination with metformin and SGLT-2 inhibitor (SGLT-2i) or GLP1 receptor agonist.

**Table 3:** Laboratory parameters in patients with active, controlled and cured disease. Data are expressed as median and interquartile range (IQR). The numbers in bold indicate significant values ( p < 0.05). IGF-I: insulin-like growth factor I; ULN: upper limit of normality; GH: growth hormone; s-Na: serum sodium; s-K serum potassium; p-Osm: plasma osmolality; u-Osm: urine osmolality

Biochemical parameters	Overall (*n* = 69)	Active disease (*n* = 22) (a)	Controlled disease (*n* = 33) (b)	Cured disease (*n* = 14) (c)	(a) vs. (b) *p*-value	(a) vs. (c) *p*-value	(b) vs. (c) *p*-value
IGF-I, *µg/L*	197.5 (150.4-249.4)	336.0 (256.0-519.9)	162.9 (134.4–201.0)	173.5 (141.4–195.0)	**< 0.001**	**< 0.001**	0.577
IGF-I / ULN	0.84 (0.63–1.05)	1.50 (1.08–1.79)	0.69 (0.60–0.86)	0.68 (0.56–0.82)	**< 0.001**	**< 0.001**	0.991
GH, *µg/L*	1.22 (0.50–2.45)	2.23 (1.24–4.39)*	0.75 (0.55–1.57)^a^	1.08 (0.46–1.67)	**0.001**	**0.008**	0.792
s-Na, *mmol/L*	140 (138–141)	140 (139–141)	139 (138–141)	141 (138–141)	0.234	0.817	0.235
s-K, *mmol/L*	4.2 (4.0-4.4)	4.1 (4.0-4.3)	4.1 (4.0-4.3)	4.3 (4.1–4.4)	0.869	0.174	0.210
Blood glucose, *mg/dL*	96 (83–113)	106 (91–133)	101 (88–113)	86 (80–94)	0.582	**0.036**	**0.048**
HbA1c, *mmol/mol*	40 (37–45)	43 (35–45)	43 (38–47)	36 (34–40)	0.464	0.082	**0.004**
Creatinine, *mg/dl*	0.78 (0.68–0.90)	0.76 (0.59–0.90)	0.76 (0.68–0.91)	0.81 (0.72–0.85)	0.731	0.548	0.861
p-Osm, *mOsm/kg*	287 (283–289)	287 (285–289)	286 (281–289)	287 (283–293)	0.290	0.933	0.456
u-Osm, *mOsm/kg*	653 (361–853)	603 (387–809)	597 (293–802)	549 (477–638)	0.197	0.110	0.612

*Evaluated in 19 patients

^a^Evaluated in 26 patients

### BIVA

Bioimpedance characteristics are presented in Supplementary Table 1. Patients with cured disease had significantly lower percentages of TBW and higher Rz compared to the other patients. A significant negative correlation was observed between IGF-I and Rz and Rz/H (*r* = −0.276, *p* = 0.022, *r* = −0.252, *p* = 0.037, respectively) while no correlation was found between GH or IGF-I levels and TBW, ECF and ICF.

**Fig. 2:**
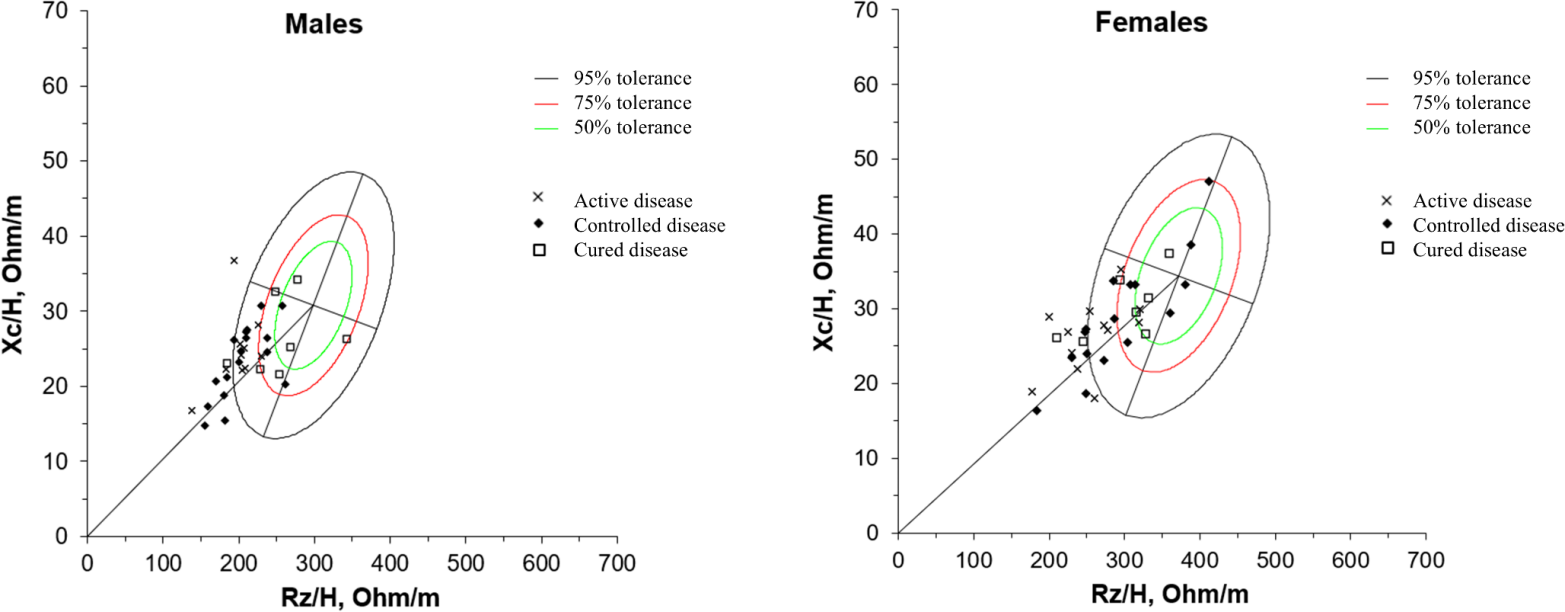
Mean vectors of acromegaly patients, divided by sex, relative to the 50th, 75th, and 95th percentiles tolerance ellipses of the healthy Italian reference population. Rz: resistance; Xc: reactance; H; height

Figure 2 displays the vectors of the patients relative to the 50th, 75th, and 95th percentiles tolerance ellipses of the healthy Italian reference population. As evident, the vast majority of subjects with either active or controlled disease were located in the lower left quadrant, out of the 75% tolerance ellipses, indicating overhydration compared to the Italian population, and this finding was confirmed in both sexes (*p* < 0.0001 at Hotelling’s T2 test for each group).

**Fig. 3:**
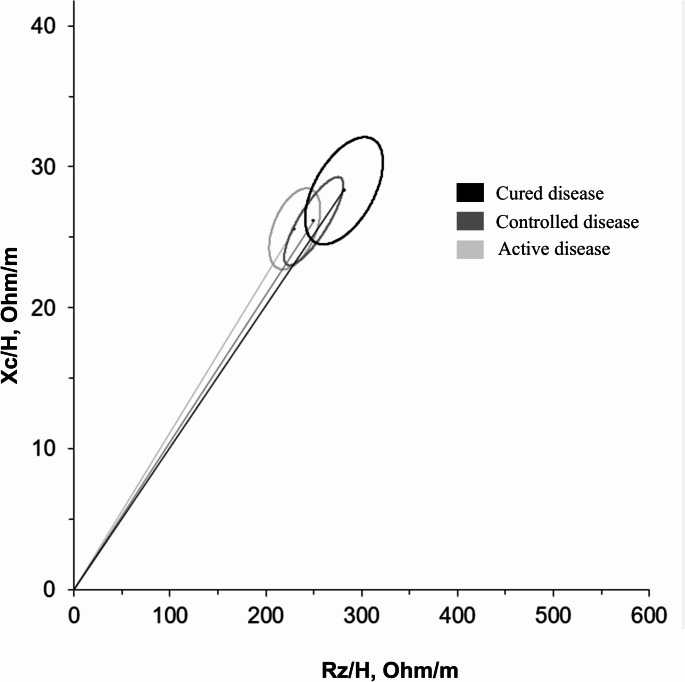
Mean Biavectors with 95% confidence ellipses of acromegaly patients according to hormonal status (active, medically controlled or cured). Rz: resistance; Xc: reactance; H; height

BIVA confidence analysis demonstrated that there were no differences between patients with active and controlled disease (*p* = 0.363), as the Biavectors were overlapped (Fig. 3). On the other hand, the Biavector of patients with cured disease was significantly longer compared to that of patients with active disease (*p* = 0.016), although no difference was observed when compared to patients with controlled disease (*p* = 0.308).

Finally, BIVA confidence analysis revealed that even cured patients had a shorter Biavector compared to the healthy reference population (data not shown), suggesting a state of overhydration. However, this difference was statistically less pronounced (*p* = 0.042 for males, *p* = 0.002 for females) than that identified for the other two groups.

## Discussion

The results of our study show that patients with either active or medically controlled acromegaly exhibit significant overhydration compared to a reference healthy population, consistent with the systemic effects mediated by GH and IGF-I. In contrast, patients with cured disease show a significantly longer Biavector than those with active disease, suggesting reduced fluid retention; however, their Biavector remains distinct from that of the Italian reference population.

The sodium and water-retaining effect of the somatotropic axis has been recognized for decades, first identified in rats and later confirmed in human beings [9, 29]. As of today, however, the pathophysiological mechanism has not been fully elucidated yet and several direct and indirect pathways have been proposed, since both GH and IGF-I have specific receptors at the renal level [10]. Specifically, increased sodium reabsorption appears to depend on hyperactivation of the epithelial sodium channel (ENaC) in the collecting duct, resulting in an increase of TBW and ECF [30]. Conversely, an action at the level of the proximal convoluted tubule has been apparently ruled out [30, 31].

In our study, we observed a significant negative correlation between IGF-I and both Rz and Rz/H. Considering that a reduction of these BIVA variables generally indicates an increase in TBW [26, 27], our data confirm that somatotropic hypersecretion may be directly involved in the pathophysiology of fluid overload. Moreover, among the proposed indirect mechanisms of action, involvement of the renin-angiotensin-aldosterone system, inhibition of atrial natriuretic peptide, as well as interaction mediated by AVP or insulin have been suggested [10].

Studies concerning the hydration status in patients with acromegaly are mostly outdated and have certainly confirmed a trend towards TBW and ECF overload [9, 10, 29]. Although isotope dilution remains the gold standard for assessing water distribution in the body [14, 15], science is actively seeking alternatives to reduce sanitary costs and patients’ exposure to radiation. In this regard, BIVA analysis employs a small, portable device and represents a considerably less expensive alternative. Several studies in the past have compared the accuracy of BIA with other methods for assessing body composition, such as dual-energy X-ray absorptiometry (DXA), finding a very high degree of correlation [32, 33]. Of note, this finding has also been recently confirmed in the acromegalic population [22] and such patients were shown to have increased TBW and ECF compared to controls [21, 23].

If the sodium and water-retaining action of the somatotropic axis is mediated primarily by GH and IGF-I, it is reasonable to expect that patients with either medically controlled or cured disease would have a lower tendency towards fluid accumulation compared to those with active disease. In our study, however, no difference in terms of hydration status between patients with active and controlled disease was observed and both groups presented a significant fluid overload compared to a reference healthy Italian population. In contrast, only patients with cured disease showed a reduced hydration status compared to patients with active disease, although no difference was observed with medically controlled patients.

In this regard, it is important to note that patients in the latter group were significantly older than those in the other groups. Older individuals often have more comorbidities and are at greater risk of altered hydration status. Specifically, these patients may experience a decrease in maximal renal water excretion, decreased eGFR, and increased secretion of AVP, while also exhibiting reduced thirst perception and impaired urine concentration ability [34]. However, no significant differences in renal function or u-Osm were observed in our cohort of medically controlled patients compared to the other groups. Therefore, it is possible that the older age in the medically controlled disease group may have both attenuated and exacerbated the impact of GH/IGF-I reduction on water balance in this group [34, 35]. Nonetheless, when comparing individuals of the same age, subjects with cured disease exhibited a longer Biavector compared to those with active disease, indicating a reduced fluid status. Additionally, the number of patients on diuretic therapy (loop and thiazide diuretics) was low and not significantly different across the three groups.

In the study by Lopes et al. [22], similarly, no significant differences in body composition were identified between patients with either active or medically controlled disease. In that study, however, a multifrequency BIA was used and the aim was to assess specifically the quantity of lean or fat mass, while BIVA analysis was not performed.

Regarding the BIVA analysis, it is worth noting that we compared our population data with the normal reference ellipses first proposed by Piccoli et al. in 1995 [19]. While a more recent study redefined these ellipses based on a larger sample, showing a general leftward shift in the mean vectors on the RXc graph [36], it focused on a population aged 18–65 years. In contrast, nearly half of the subjects in our cohort (*n* = 27) were over 65, which aligns more closely with the broader age range (15–85 years) of Piccoli’s original reference population [19].

Moreover, nearly all patients with medically controlled disease in our cohort were treated with somatostatin receptor ligands, which are generally considered the first-line medical treatment for acromegaly [37]. Although not previously reported in the context of acromegaly, studies conducted initially in rabbits [38, 39] and subsequently in humans [40] have shown that treatment with octreotide is capable of stimulating water and sodium reabsorption predominantly through an action at the level of the small intestine. Indeed, these drugs are also used for the treatment of refractory secretory diarrhea and dumping syndrome [41]. In this regard, therefore, a potential confounding effect of the same medical therapy on the hydration status cannot be completely ruled out.

Finally, patients with cured disease were less affected by diabetes mellitus compared to the other subjects, and as a consequence, they had significantly lower blood glucose levels. This finding seems to confirm that biochemical control of the disease has a positive metabolic impact, potentially leading to the remission of pre-existing diabetes mellitus [42, 43]. Furthermore, diabetes mellitus itself may lead to volume overload, and its resolution or better management, especially through the use of SGLT-2i, has been shown to improve BIVA parameters [18]. In any case, it is relevant to note that in our cohort diabetes mellitus was well-controlled in almost all patients and only 5 individuals were treated with SGLT-2i, thus minimizing the impact of this comorbidity on the results obtained.

Our study presents some strengths and limitations. One strength is the sample size, significantly larger than previous studies [22, 23]. Additionally, unlike other studies, we performed BIVA, which, as previously mentioned, allows for the accurate assessment of hydration status even in unhealthy individuals, based directly on bioimpedance parameters. Finally, we included a subgroup of subjects with cured disease in the absence of medical therapy, and our cohort was quite homogeneous in terms of both sex distribution and disease activity. The major limitation of the study concerns its cross-sectional design, in which patients were evaluated at a single point in their disease history. Additionally, information on water and salt intake was collected through direct inquiry, which may render it less reliable. It is important to note, however, that current evidence does not clearly associate variations in BIVA parameters with differing levels of routine fluid or salt intake. This contrasts with the acute consumption of beverages or food, which can significantly influence bioimpedance results [44, 45]. Finally, in patients with cured acromegaly, the somatotropic axis was not reassessed to exclude potential GHD, which could otherwise contribute to a reduction in body water content [46]. However, only 2 patients among them presented concomitant anterior panhypopituitarism, and only one individual had IGF-I values below the age-normal limit, making it unlikely that this significantly affected our results.

## Conclusion

The findings of our study demonstrate that, according to BIVA analysis, patients of both sexes with either active or medically controlled acromegaly present a marked overhydration compared to a healthy reference Italian population. Conversely, patients with cured disease show a reduced level of hydration compared to those with active disease; however, their Biavector remains shorter than that of the reference population, suggesting a residual degree of fluid retention. This observation, though partly explainable by the smaller sample size of cured patients, might suggest a long-term persistence of the known effects of GH and IGF-I even after biochemical remission.

Considering that increasing attention is being given to new tools for accurately assessing disease compensation in acromegalic patients [47, 48], further studies are necessary to evaluate the possibility of integrating BIVA analysis into the routine assessment of patients affected by this condition, as well as to confirm the persistence of fluid overload in patients with cured disease.

## Electronic supplementary material

Below is the link to the electronic supplementary material.

Supplementary Material 1

## Data Availability

The data sets generated during and/or analyzed during the current study are not publicly available but are available from the corresponding author on reasonable request.
