# Supplementary material for: Evaluation of fluid status in patients with acromegaly through bioelectrical impedance vector analysis: a cross-sectional study
Source: J Endocrinol Invest. 2025 Feb 15;48(5):1185–95. doi: 10.1007/s40618-025-02541-4 (PMC12049396; doi:10.1007/s40618-025-02541-4)
Supplement: Supplementary file 1 — Supplementary Material 1 [file 40618_2025_2541_MOESM1_ESM.docx]

| **Bioimpedance parameters** | **Overall**  **(n=69)** | **Active disease**  **(n=22)**  **(a)** | **Controlled disease**  **(n=33)**  **(b)** | **Cured disease**  **(n=14)**  **(c)** | **(a) vs (b)**  **p-value** | **(a) vs (c)**  **p-value** | **(b) vs (c)**  **p-value** |
| --- | --- | --- | --- | --- | --- | --- | --- |
| Rz, *Ohm* | 410.0 (347.4-473.2) | 370.1 (343.8-435.4) | 408.5 (345.4-471.8) | 463.5 (428.5-543.4) | 0.311 | **0.003** | **0.038** |
| Xc, *Ohm* | 44.4 (39.8-49.7) | 43.6 (39.8-45.4) | 45.9 (36.4-49.7) | 47.1 (42.9-54.2) | 0.594 | **0.038** | 0.106 |
| Rz/H, *Ohm/m* | 237.7 (203.6-288.8) | 225.5 (201.9-260.7) | 237.7 (199.1-285.8) | 286.0 (248.3-327.6) | 0.381 | **0.006** | 0.077 |
| Xc/H, *Ohm/m* | 26.2 (22.4-29.6) | 26.4 (22.2-28.2) | 26.2 (21.1-29.7) | 26.6 (25.2-32.6) | 0.993 | 0.194 | 0.245 |
| PhA (°) | 6.2 (5.4-6.9) | 6.5 (5.8-6.9) | 6.2 (5.4-6.7) | 5.5 (4.9-7.0) | 0.246 | 0.199 | 0.522 |
| TBW, *L* | 46.4 (37.3-53.9) | 49.8 (39.0-56.2) | 44.9 (36.4-54.3) | 39.5 (37.0-49.7) | 0.531 | 0.144 | 0.500 |
| TBW (%) | 61.1 (55.1-67.0) | 62.4 (55.8-67.2) | 64.1 (55.9-68.3) | 55.7 (52.0-60.2) | 0.637 | **0.007** | **0.003** |
| ECF, *L* | 20.1 (17.5-23.6) | 22.1 (17.5-23.6) | 20.1 (18.1-23.5) | 19.2 (17.0-24.0) | 0.764 | 0.685 | 0.617 |
| ECF (%) | 44.9 (41.9-48.8) | 43.9 (41.8-46.6) | 44.9 (42.4-48.8) | 48.1 (41.4-51.6) | 0.271 | 0.173 | 0.493 |
| ICF, *L* | 25.2 (19.1-31.9) | 28.4 (20.4-32.7) | 23.9 (28.9-32.0) | 24.5 (18.1-28.6) | 0.465 | 0.292 | 0.825 |
| ICF (%) | 55.1 (51.2-58.1) | 56.2 (53.4-58.2) | 55.1 (51.3-57.8) | 51.9 (48.4-58.6) | 0.271 | 0.173 | 0.493 |
